# Biological control and management of *Dalbulus maidis* (Hemiptera: Cicadellidae) with egg parasitoids: a review

**DOI:** 10.1093/jisesa/ieag036

**Published:** 2026-04-23

**Authors:** Gustavo Moya-Raygoza

**Affiliations:** Departamento de Botánica y Zoología, CUCBA, Universidad de Guadalajara, Zapopan, Jalisco, Mexico

**Keywords:** Hymenoptera, insect pest, insect ecology, maize, integrated pest management

## Abstract

The corn leafhopper *Dalbulus maidis* (DeLong & Wolcott) has become one of the most important pests on maize throughout the American continents. Nymphs and adults of *D. maidis* are efficient vectors of maize pathogens that produce corn stunt diseases. Biological control using predators, entomopathogenic fungi, and parasitoids against *D. maidis* nymphs and adults are reviewed, giving special attention to their effect on transmission biology. Egg parasitoids of the corn leafhopper are becoming the best alternative to control *D. maidis* populations. The present review identifies the most common and abundant egg parasitoids in maize fields throughout tropical America, characterizing them both morphologically and molecularly. These parasitoids are *Anagrus virlai* Triapitsyn (Hymenoptera: Mymaridae) and *Paracentrobia subflava* (Girault) (Hymenoptera: Trichogrammatidae). The development and behavior of these 2 species are also reviewed, and a new methodology is presented for attracting and releasing these parasitoids in maize fields. This new methodology is based on the conservation biological control strategy of edge management, combined with the augmentative biological control strategy of egg parasitoid release. The effect of diet on adult parasitoid fitness is also analyzed. Finally, the use of volatile organic compounds to improve the efficiency of these egg parasitoids in finding *D. maidis* eggs is reviewed. Further studies are suggested and gaps in knowledge are highlighted and analyzed throughout the review.

## Introduction

The corn leafhopper *Dalbulus maidis* (DeLong & Wolcott) is a maize specialist pest (Nault and DeLong 1980) that is causing increased damage while expanding its range in the American continents. Over the past 5 yr, it has become one of the most important pests of maize throughout the Americas (Moya-Raygoza 2026). In the United States, this insect has expanded to new geographical areas, and for first time it has been documented along the upper Midwest, in Illinois, Indiana, Iowa, Kansas, Kentucky, Michigan, Minnesota, Missouri, Nebraska, and Wisconsin (Lagos-Kutz et al. 2025). In Brazil, this insect reduced maize production by about 40% in 2020–2022 (Canale et al. 2023); and in Argentina, maize production was reduced by 77% in 2023–2024 (Cabrini et al. 2024). In Mexico, little is known about maize losses by this insect pest during the past 5 yr. Nymphs and adults of *D. maidis* primarily cause damage through transmission of corn stunt diseases including corn stunt spiroplasma, caused by *Spiroplasma kunkelii* Whitcomb; maize bushy stunt phytoplasma, caused by *Candidatus* Phytoplasma asteris; and infection with maize rayado fino virus (Nault 1980). These 3 pathogens are transmitted in a persistent propagative manner (Moya-Raygoza 2026).

Chemical control has been used against corn leafhopper nymphs and adults. In the United Sates and Brazil, most insecticides against *D. maidis* adults are applied when maize plants are young (Moya-Raygoza 2026). In Brazil, insecticides have been used intensively in the past against the corn leafhopper. However, resistance against pyrethroids and neonicotinoid insecticides has recently been found, and this could explain the documented failure in controlling *D. maidis* in Brazil (Machado et al. 2024).

Predators, entomopathogenic fungi, and parasitoids are compatible with a healthy environment and could be used for biological control of *D. maidis* nymphs or adults throughout the American continents. Forty species of arthropod predators that attack nymphs or adults of *D. maidis* have been documented throughout the Americas (Casuso et al. 2025). Predators can efficiently control leafhoppers that transmit plant pathogens in a persistent manner, affecting feeding behavior and the prevalence of the vector (Tholt et al. 2018). *Gonatopus bartletti* Olmi (Hymenoptera: Dryinidae) is a predator and a parasitoid of the corn leafhopper. Predators such as *G. bartletti* that affect the survival of *D. maidis* bearing *S. kunkelii* could be an effective biological alternative to chemical pesticides (Moya-Raygoza et al. 2008). Entomopathogenic fungi of the genera *Beauveria*, *Metarhizium*, and *Cordyceps* have been investigated to control *D. maidis* adults in the Americas. However, their use to control *D. maidis* adults bearing plant pathogens is limited because of the delay in killing the vector, low rate of mortality of infected vectors, and barrier brochosomes produced by the corn leafhopper (Lopes et al. 2025). Nymphs and adults of *D. maidis* are parasitized by 6 species of Hymenoptera (Dryinidae), 3 species of Diptera (Pipunculidae), and 1 species of Strepsiptera (Halictophagidae) in the Americas (Moya-Raygoza 2019).

Egg parasitoids are efficient biological control agents against insect pests (Mills 2010, Sann et al. 2018). Eggs of *D. maidis* are parasitized by several micro-hymenopteran (Mymaridae and Trichogrammatidae) species in maize fields throughout Latin America (Moya-Raygoza 2019). Although a long list of egg parasitoids of *D. maidis* exists, the highest percentages of parasitism, throughout the Americas, have been reported by the egg parasitoids *Anagrus virlai* Triapitsyn (Mymaridae) and *Paracentrobia subflava* (Girault) (Trichogrammatidae). In corn fields, it is very common to find these 2 species within the same corn field parasitizing *D. maidis* eggs. In Mexico, both parasitoid species co-occur in maize fields and more than 80% of experimental maize leaves bearing *D. maidis* eggs were found to be parasitized by these 2 egg parasitoid species (Moya-Raygoza and Muñoz-Urias 2025). In South America, *A. virlai* is the most abundant egg parasitoid of *D. maidis* in maize fields (Luft Albarracin et al. 2017, Moya-Raygoza et al. 2012). Biological control using *A. virlai* and *P. subflava* is a good alternative method for controlling the populations of *D. maidis*, because (i) their highest percentage of parasitism is found in field conditions; (ii) both species are generalist parasitoids; (iii) both species attack *D. maidis* eggs before the emergence of nymphs, which are able to transmit all 3 major maize pathogens; and (iv) both species are pro-ovigenic, meaning that females are born with most of their eggs already mature.

The aim of the present review is to provide a synthesis of research in biological control of *D. maidis* nymphs and adults and management of this pest with egg parasitoids. Predators, entomopathogenic fungi, and parasitoids of *D. maidis* nymphs and adults are included, along with their interactions with *D. maidis-*transmitted plant pathogens. Egg parasitoids of *D. maidis* are biological control agents; therefore, this review is focused on *A. virlai* and *P. subflava*, providing precise information on their identification, development, and behavior. A new methodology for producing and releasing these 2 parasitoid species in maize fields is described in this review. This methodology has been established as an effective way to control *D. maidis* in field conditions, using egg parasitoids that inhabit maize edge habitats (conservational biological control) and releasing these egg parasitoids (augmentative biological control). Additionally, future biological control strategies are also presented, such as the use of volatile organic compounds (VOCs) that could improve the efficiency of these egg parasitoids in controlling *D. maidis*.

## Biological Control of Nymphs and Adults

### Predators

Nymphs and adults of *D. maidis* are predated within maize fields throughout the Americas by 40 different species of arthropods belonging to the orders Araneae, Dermaptera, Hemiptera, Coleoptera, Neuroptera, Diptera, and Hymenoptera (Casuso et al. 2025). The orders of arthropods that attack the corn leafhopper have been reported in general as predators of leafhoppers and planthoppers (Döbel and Denno 1994, Olivier et al. 2012). Predators are generalists and attack different species of leafhoppers and planthoppers, increasing predator abundance when there is an increase in leafhopper abundance (Döbel and Denno 1994). Predators have been reported as efficient biological control agents against vectors of plant pathogens with persistent transmission, in which the acquisition period entails about 24 h or more to acquire the plant pathogen. In addition, predation has been shown to affect the dynamics of acquisition of the pathogen by the vector. For example, the predator spider *Tibellus oblongus* (Walckenaer) altered the feeding behavior of the leafhopper *Psammotettix alienus* (Dahlbom), the vector of the wheat dwarf virus, which is transmitted in a persistent circulative manner. This altered feeding behavior caused by the presence of the predator affected the acquisition period of the virus, limiting the spread of the virus by the vector (Tholt et al. 2018). Similar results were found in insect predators that attacked the aphid *Rhopalosiphum padi* (L.), which transmits the cereal yellow dwarf virus in a persistent circulative manner; specifically, the predator presence stimulated vector movement, and thereby reduced pathogen prevalence, reducing the efficiency of pathogen transmission (Long and Finke 2015).

*Dalbulus maidis* has a persistent propagative pattern of transmission and needs about 48 h to complete acquisition of any of the 3 pathogens it transmits. Therefore, predators may reduce pathogen prevalence in maize fields as their presence is likely to affect *D. maidis* feeding behavior. In addition, pathogen acquisition may affect the survival of *D. maidis* in the presence of a predator: Experiments performed under controlled conditions found that the survival of *D. maidis* adults exposed to predation by *G. bartletti* was lower when the leafhoppers were infected with *S. kunkelii* for a total duration of 10 or 20 d compared with leafhoppers that were infected with *S. kunkelii* for a total duration of 2 d. In other words, being infected with the pathogen for a longer period increased leafhopper susceptibility to predation by *G. bartletti* (Moya-Raygoza et al. 2008), introducing the possible dynamic of evolutionary pressure, exerted by predators, for *D. maidis* to avoid acquisition of pathogens. *Gonatopus bartletti* is a common and abundant predator of *D. maidis* in maize fields, and it preys on nymphs and adults at rates of 22% and 26%, respectively, under caged controlled conditions (Rios-Reyes and Moya-Raygoza 2004). Further studies could investigate plant pathogen prevalence and acquisition in the context of using predators to control *D. maidis.* Also, predator communities that inhabit maize edges, particularly during the dry season, could be investigated. Maize edges have green foliage year-round, providing stable habitat for leafhoppers and parasitoids (Moya-Raygoza 2026).

### Entomopathogenic Fungi

Entomopathogenic fungi of the genera *Beauveria*, *Metarhizium*, and *Cordyceps* have been tested to control *D. maidis* adults in tropical America. However, the delay in killing the vector, low rate of mortality of the vector, and barriers by the vector against the fungi limit the use of entomopathogenic fungi to control vector transmission of plant pathogens with persistent propagative transmission. In Argentina, it was found that the average time to mortality for *Beauveria bassiana* (Balsamo) Vuillemin was 8.6 d, and the mortality rate of *D. maidis* adults sprayed with this fungus under laboratory conditions only reached 49% (Toledo et al. 2007). In corn leafhopper adults sprayed with *Metarhizium anisoplae* (Metsch.) Sorokin, the average time to mortality was 10.5 d and only reached 40% mortality under laboratory conditions in Mexico (Ibarra-Aparicio et al. 2005). Recently, in Brazil, an even lower mortality rate—35%—was reported for *D. maidis* adults exposed to maize plants inoculated with *Beauveria* spp. or *Metarhizium* spp. under caged conditions (Lopes et al. 2025). This low mortality is due to brochosomes and self-cleaning behavior of the leafhopper—defenses against fungus infection that prevent adhesion of conidia to the *D. maidis* cuticle, decreasing insect mortality (Lopes et al. 2025). There is no information on the efficiency of entomopathogenic fungi against *D. maidis* eggs.

On the other hand, fungi may play positive maize-protective roles beyond pest mortality. Probing behavior of *D. maidis* is affected negatively by *Cordyceps javanica* (Friederichs and Bally), mainly 48 h after its application, and this may reduce the rate of acquisition of plant pathogens by *D. maidis* (Maluta et al. 2023). These authors found that insects treated with the fungus spend less time performing probes and spend a shorter duration ingesting phloem sieve than do the untreated insects. However, a 2025 study found that changes in *D*. *maidis* feeding behavior induced by *B. bassiana* infection were insufficient to reduce the incidence and severity of corn stunt diseases and grain yield losses in maize (Oliveira et al. 2025). These authors found that the survival of *D. maidis* adults is not affected by application of *B. bassiana* on leafhoppers bearing *S. kunkelii* or phytoplasma. Similar results were found when *D. maidis* adults received an application of *M. anisoplae*: the survival of *D. maidis* adults bearing *S. kunkelii* was not affected by application of the fungus (Moya-Raygoza et al. 2008).

### Parasitoids

*Dalbulus maidis* parasitoids develop within nymphs or adults of this vector. These are holometabolous insects that belong to the orders Hymenoptera (Dryinidae), Diptera (Pipunculidae), and Strepsiptera (Halictophagidae) in the tropical Americas (Moya-Raygoza 2019). Once larvae have completed development within *D. maidis*, they emerge, killing the leafhopper, and after emergence, they pupate outside the host. The parasitoid needs about 12 d to develop from egg to the last larval instar in the case of the dryinid *G. bartletti* (Rios-Reyes and Moya-Raygoza 2004). This species is found throughout the American continents, and in Mexico, reached the highest percentage of parasitism, with 24% parasitism in maize fields (Moya-Raygoza et al. 2004). No information exists about the number of days needed for larval development of Pipunculidae or Halictophagidae parasitoids within *D. maidis*.

Development of nymph or adult parasitoids and reproduction of any of the 3 plant pathogens can occur within a *D. maidis* host. Experiments conducted with *D. maidis* parasitized by *G. bartletti* and bearing *S. kunkelii* found that larval development of the parasitoid was successfully completed and survival of *S. kunkelii* in *D. maidis* was negatively affected by the parasitoid larvae (Moya-Raygoza et al. 2006). These results suggest that the parasitoid can reduce the presence of bacterial plant pathogens within *D. maidis*. However, future studies are needed to investigate *D. maidis* feeding behavior and dispersal when bearing both plant pathogens and larval parasitoids, simultaneously developing within the corn leafhopper.

## Host Pest: *Dalbulus maidis*

### *Dalbulus maidis* and Its Eggs

Precise and fast identification of an insect pest is needed to target control strategies. The corn leafhopper *D. maidis* is a hemimetabolous insect of the family Cicadellidae and order Hemiptera. *Dalbulus maidis* adults are small insects, 3.7 to 4.3 mm in length, with variable color. Adults can be easily identified using these 3 morphological characters: (i) adult head with 2 large black anterior spots, (ii) aedeagus of males with divergent apical process, and (iii) seventh sternum of females deeply notched (Moya-Raygoza 2026). In addition, *D. maidis* definitive identification can be performed using molecular markers. Molecular identification can be made using DNA amplification of the gene encoding cytochrome C oxidase subunit I (COI). The sequences of *D. maidis*-specific primers are: dalCOI fwd: 5′ TAG CTC AAC CTG GGT CGT TT, and dalCOI rev: 5′ TGG TAT AGG ATT GGG TCA CCA (Palomera et al. 2012).

*Dalbulus maidis* lays most eggs in clusters on the midrib of maize leaves (Heady et al. 1985, Moya-Raygoza 2016). Females insert eggs singly into maize tissue with the ovipositor (Nault 1998). The development of *D. maidis* is temperature dependent (Madden et al. 1986). At 25 °C, Nault (1998) found that the egg stage lasts about 9 d and once the eggs complete this developmental stage, nymphs emerge. Precise emergence of nymphs from Mexican *D. maidis* populations was determined in 2024: at 25 ± 2 °C, it was found that on average, nymphs begin to emerge at day 9.70 (SE = 0.13) and end their emergence on average at day 10.92 (SE = 0.12) (G. Moya-Raygoza, personal observation).

## Egg Parasitoids: *Anagrus virlai* and *Paracentrobia subflava*

### Identification

Morphological characters and molecular markers are important for precise identification of *A. virlai* and *P. subflava*, and precise identification is important for their use as biological control agents. *Anagrus virlai* Triapitsyn has been misidentified previously as *Anagrus breviphragma* Soyka, and later as *Anagrus incarnatus* Haliday. *Anagrus virlai* females have a length of 0.40 to 0.60 mm, body mostly light brown, clava with 5 multiporus plate sensilla, forewing disc with several rows of setae with one complete row originating behind the apex of venation and about 1 to 2 irregular rows in the broadest part of the disc (Triapitsyn et al. 2019). Polymerase chain reaction has been employed to amplify a region of the mitochondrial cytochrome c oxidase subunit I gene (COI gene). This method can be conducted using the following primers: LCO1490 (5′-GGTCAACAAATCATAAAGATATTGG-3′) and HCO2198 (5′-TAAACTTCAGGGTGACCAAAAAATCA-3′ (Triapitsyn et al. 2019).

*Paracentrobia subflava* (Girault) has also been misidentified; as *Paracentrobia tapajosae* (Viggiani). The *Paracentrobia subflava* female body is 0.65 mm, has opaque lemon yellow color, forewings broad but narrow, with cilia consisting of 8 to 14 longitudinal lines counted along the widest part (Girault 1911). The forewing has a dark spot behind the stigma vein on the disc, and it is less pubescent than *P. tapajosae* (Moya-Raygoza and Triapitsyn 2017). Combined taxonomic, morphological, and molecular characters indicated that *P. tapajosae* is considered a junior synonym of *P. subflava* (Torres-Moreno et al. 2022). The sequence of the mitochondrial COI gene can be used for molecular characterization of *P. subflava*. The primers used to amplify the COI mitochondrial gene are: C1-J-2183: 5′-CAA CAT TTA TTT TGA TTT TTT GG-3′ (forward) and TL2-N-3014: 5′-TCC AAT GCA CTA ATC TGC CAT ATT A-3′ (reverse) (Torres-Moreno et al. 2022).

### Development

*Anagrus virlai* and *P. subflava* adults find, parasitize, and develop within *D. maidis* eggs and emerge as adults. When *D. maidis* feeds on plants and lays eggs, the plant is damaged and releases volatiles (herbivore-induced plant volatiles). These volatiles provide the parasitoid with precise information to find leaves with leafhopper eggs, increasing host searching efficiency (Moya-Raygoza 2016). Twenty different volatiles were found to be produced in the landrace maize and hybrid maize after *D. maidis* injury; however, a higher abundance of volatiles is released by landrace maize than by hybrid maize, according to data from Argentina, in laboratory conditions (Coll-Aráoz et al. 2020). These volatiles, emitted following damage by *D. maidis*, attracted more *A. virlai* egg parasitoids to the landrace maize than to the hybrid maize, under laboratory conditions (Coll-Aráoz et al. 2020). On rice, the rice brown planthopper *Nilaparvata lugens* (Stål), another piercing-sucking insect, induces 20 different volatile compounds to attract *Anagrus nilaparvatae* Pang and Wang (Lou et al. 2005b). These same authors found that treating the plant wound with jasmonic acid induced greater volatile emissions that attracted *A. nilaparvatae* to parasitize the *N. lugens* eggs (Lou et al. 2005a). In general, stimulation of herbivore-induced plant volatiles is a current biological strategy being proposed to control insect pests in agriculture (Turlings and Erb 2018).

Once the female finds *D. maidis* eggs, she will start to lay her parasitoid eggs within these host eggs. Then, larval parasitoids start to develop within the *D. maidis* eggs. Eggs parasitized by *A. virlai* turn red in color and eggs parasitized by *P. subflava* turn black. The development of the parasitoid is temperature dependent. Once the parasitoids complete development, they emerge from the eggs as adults. *Anagrus virlai* adults emerge before *P. subflava* adults. *Anagrus virlai* adults start to emerge on average at 16.74 (SE = 0.24) d and *P. subflava* adults start to emerge on average at 23.34 (SE = 0.32) d (Moya-Raygoza and Muñoz-Urias 2025). Knowing the emergence period of *A. virlai* and *P. subflava* contributes to determining a precise date for release of these parasitoids in a corn field to control *D. maidis* populations.

### Behavior

In pro-ovigenic parasitoids, females are born with most of their eggs already mature (Jervis et al. 2001). Adult parasitoids of *A. virlai* are pro-ovigenic (Hill et al. 2020), as are adult parasitoids of *P. subflava* (Torres-Moreno and Moya-Raygoza 2025). Pro-ovigenic parasitoid species have reproductive abilities that allow them to exploit all available hosts at a low cost of egg production (Cusumano et al. 2022). In addition, both *A. virlai* and *P. subflava* are generalist parasitoids (Triapitsyn et al. 2019, Hill et al. 2023, Torres-Moreno and Moya-Raygoza 2024). Both parasitoid species parasitize eggs of Deltocephalinae leafhoppers on wild green perennial grasses on the maize edges during the off-maize season (dry season) (Moya-Raygoza 2024). Therefore, maize edges play an important role in integrated pest management as they serve as habitat for conserving leafhopper egg parasitoids. Once maize is planted during the maize-growing wet season, adult parasitoids start to disperse from the edges to the new planted maize to parasitize the eggs of *D. maidis* immigrants (Moya-Raygoza 2024).

## Agricultural Application

### A Protocol for Producing and Releasing Egg Parasitoids, Using Sentinel Maize Plants, to Control *D. maidis*

**Fig. 1.**
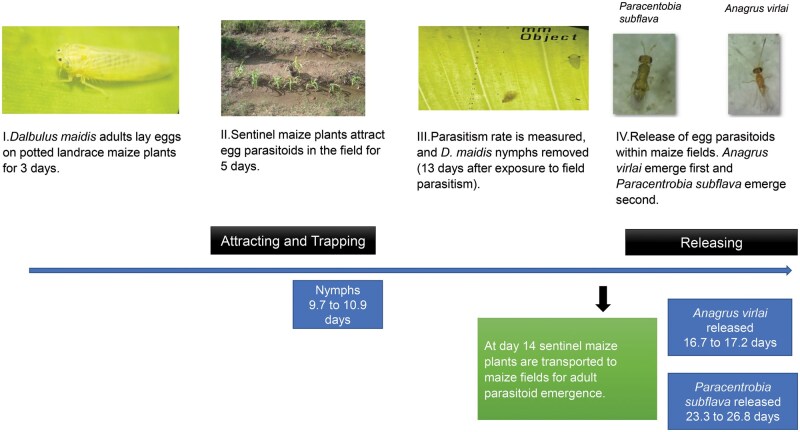
Scheme of producing *D. maidis* eggs (phase I), attracting and trapping their egg parasitoids from the maize edges (phase II), calculation of parasitism rate and removal of nymphs (phase III), and releasing the trapped parasitoids within the maize field (phase IV).

A new methodology for the biological control of *D. maidis* using sentinel maize plants is presented in this review. This methodology is based on observations and results obtained from studies conducted under field and laboratory conditions. This methodology is divided into 4 phases. Phases I and II are focused on attracting and trapping *A. virlai* and *P. subflava* living in the maize edges bordering the target field, using eggs of *D. maidis* laid on sentinel maize plants. Phases III and IV are focused on releasing the trapped egg parasitoids within the maize field (Fig. 1).

#### Phase I. *Dalbulus maidis* Eggs on Sentinel Landrace Maize Plants

A large number of *D. maidis* adults need to be reared. Corn leafhopper adults are found on the foliage of maize plants in most maize fields and their identification can be performed using the characters indicated above. Rearing cages with potted maize plants are used to rear *D. maidis*. Once there is a large number of reared *D. maidis* adults, they are placed within leaf-cages using landrace maize at the 5-leaf stage. A total of 16 *D. maidis* adults (8 females and 8 males) are placed in each leaf cage, which is set on leaf number 4. Two maize plants can be used per pot. Maize landrace should be used because it releases more volatiles than hybrid maize when *D. maidis* feed or oviposit, and these volatiles attract *A. virlai* (Coll-Aráoz et al. 2020). *Dalbulus maidis* adults are then allowed to feed and oviposit in a rearing room at 25 ± 2 °C, 50% relative humidity, and a photoperiod of 12:12 (L:D) h for 72 h. The number of leaf-cages to use depends on the number of adults available.

After a 72-h oviposition period, adults are removed and transferred to the rearing cage, and the landrace maize plants bearing the oviposited eggs (sentinel maize plants) are transported to the maize fields to expose them to natural parasitism by *A. virlai* and/or *P. subflava*. *Dalbulus maidis* egg development time at 25 °C is about 9 d (Nault 1998), and nymph emergence occurs between 9.7 and 10.9 d (G. Moya-Raygoza, personal observation). Egg development and nymph emergence time is important to know how long eggs should be exposed to parasitism. This phase can be conducted under greenhouse conditions or outdoor conditions, but the number of days for egg development should be determined under those temperature conditions.

#### Phase II. Sentinel Maize Plants Attracting Egg Parasitoids of *D. maidis*

Potted maize plants bearing *D. maidis* eggs (sentinel plants) are transported to maize fields to attract egg parasitoids. Once the *D. maidis* adults are removed from the leaf-cages, the potted maize plants should be transferred immediately to the maize field. Maize plants infested with 30 *D. maidis* adults show an induced volatile response 24 h post-injury, and this response persists for up to 96 h (Sanches et al. 2025). The potted maize plants should be set in the outer lines of the maize fields, adjacent to the maize edges. Eggs of most sentinel maize plants are found and parasitized by egg parasitoids on the maize edges rather than within the maize field (Moya-Raygoza and Muñoz-Urias 2025). This is not surprising, as maize edges are overwinter habitats for egg parasitoids of *D. maidis* (Moya-Raygoza 2024). Grasses that grow on the edges maintain green foliage during the dry season and maintain egg parasitoids and leafhoppers, but not *D. maidis* (Moya-Raygoza and Becerra-Chiron 2014). Moreover, edges are generally not sprayed with insecticides by farmers. Therefore, in this phase, edges are considered a tool for biological control by conservation. Potted sentinel maize plants are maintained in the field for 5 d to allow natural field parasitism. These pots should receive water if needed to avoid plant wilting. Fewer days in the field will result in fewer plants found and parasitized by egg parasitoids. In a 72-h oviposition period, eggs of *D. maidis* on sentinel maize plants maintained adjacent to the maize edges during a 5-d period have been found to become more than 80% parasitized by *A. virlai* and *P. subflava* (Moya-Raygoza and Muñoz-Urias 2025).

#### Phase III. Development of Parasitized Eggs, Parasitism Rate, and Removal of *D. maidis* Nymphs

After 5 d, potted maize plants are returned to laboratory reared conditions at 25 ± 2 °C, 50% relative humidity, and a photoperiod of 12:12 (L:D) h. These plants are maintained for 8 d in a rearing room. In total, 13 d (5 in the field and 8 in the rearing room) will have passed since eggs were exposed to natural parasitism. On day 13, each leaf exposed to parasitism is reviewed to determine the percentage of parasitism by *A*. *virlai* and *P. subflava* and to remove *D. maidis* nymphs using a stereoscopic (dissecting) microscope. On the 13th day, eggs parasitized by *A. virlai* show red color and eggs parasitized by *P. subflava* show black color, and most nymphs have emerged. Emerged nymphs are removed with a brush to avoid their propagation in the next phase, when pots are returned to the field for parasitoid emergence as adults. Once the percentage of parasitism per maize leaf has been determined, those maize plants with a low percentage of parasitism can be discarded. This phase can be conducted under greenhouse conditions or outdoor conditions, but the number of days in egg development should be determined under those temperature conditions.

#### Phase IV. Releasing Egg Parasitoids within Maize Fields Infested with *D. maidis*

Potted maize plants are set within maize fields so that the adult parasitoids will emerge and establish within the maize fields. This step should be performed on day 14 in order to ensure that the parasitoids emerge there. *Dalbulus maidis* overwinter as adults, not as eggs (Larsen et al. 1992), and adults are observed first in a newly planted maize field. Corn leafhopper adults have negative binomial distribution in the field (Carmo et al. 2025), with the highest abundance of immigrant adults occurring on the edges of the maize fields, and this is where they first start to lay eggs (Foresti et al. 2022). Therefore, pots with sentinel eggs are suggested to be placed in these edge sites where immigrant *D. maidis* adults will first arrive. If the parasitized eggs show red color, the pots should remain for 1 wk in the field. If the parasitized eggs show red and black color, the pots should remain in the field for 2 wk. The *A. virlai* adults will emerge first, between days 16.74 (SE = 0.24) and 17.25 (SE = 0.24). Later, the *P. subflava* adults will emerge, between days 23.34 (SE = 0.32) and 26.89 (SE = 0.43) (G. Moya-Raygoza, personal observation). The pots with the maize plants should receive water to ensure that the plant will thrive and ensure parasitoid emergence. Once emerged, adults of these 2 species will be able to find and parasitize *D*. *maidis* eggs, because both species are pro-ovigenic. Also, these 2 species cause the nymph to die before emergence. This phase represents augmentative biological control, because parasitoids of both species are released in maize agroecosystems.

This methodology requires little cost in materials but intensive labor. Materials such as rearing cages and pots for planting landrace maize can be accessible for farmers. Labor intensity is required in collecting and maintaining *D. maidis* colonies, removing nymphs, selecting sentinel maize plants with a high level of parasitism, and transporting potted maize plants. Farmers could obtain benefits not only in maize yield but also in cost savings for avoiding the use of insecticides, human health, and ecosystem health. The rearing process is detailed in this suggested augmentative biocontrol method to facilitate further mass production of *A. virlai* and *P. subflava*. In general, this methodology can be adapted to most environmental conditions seen on the American continents. In each environmental condition, the time for *D. maidis* egg development and time for adult parasitoid emergence will be reduced if the temperature is increased, because the development of both host eggs and parasitoids is temperature dependent.

### Effect of Diet on Adult Fitness of *A. virlai* and *P. subflava*

Diets have effects on the fecundity and longevity of adult egg parasitoids. Females of *A. virlai* fed with a mixture of honey and honeydew increase their fecundity and longevity (Hill et al. 2020). In addition, the longevity of *P. subflava* adults has been shown to be higher when fed on a honey diet (Torres-Moreno and Moya-Raygoza 2024) and female *P. subflava* from mothers fed with honey exhibit larger body size (Torres-Moreno and Moya-Raygoza 2025). Honeydew is an abundant resource in agroecosystems that is produced by sucking insects that fed on the phloem of plants (Wäckers et al. 2008, Tena et al. 2013). Honeydew is not only produced by *D. maidis* but also by other leafhoppers (Pinedo-Escatel and Moya-Raygoza 2018) and planthoppers (Hemiptera: Delphacidae) (Rodríguez-Juárez et al. 2020) that inhabit within the maize field and on the maize edges. Another food resource for *A. virlai* and *P. subflava* adults is plants that produce nectar and pollen and grow within maize fields (Moya-Raygoza and Figueroa-Bautista 2021). These available food resources for *A. virlai* and *P. subflava* adults could enhance their survival and fecundity in field conditions. Further studies could be developed to enable mass production of *A. virlai* and *P. subflava* using honey to increase their longevity and fecundity.

### VOCs Compatible With Egg Parasitoids

Volatile organic compounds emitted by host plants damaged by sap-sucking herbivores are becoming another tool to enhance biocontrol using egg parasitoids. Rice plants wounded and treated with jasmonic acid increase emission of VOCs that attract the egg parasitoid *A. nilaparvatae*, which parasitizes eggs of *N. lugens*, under field conditions (Lou et al. 2005a). Under laboratory conditions, the volatiles β-caryophyllene, β-farnesene, and α-bergamotene were emitted following damage inflicted on landrace maize by *D. maidis*, and these volatiles attracted *A. virlai* (Coll-Aráoz et al. 2020). Also, β-caryophyllene, β-farnesene, *α*-bergamotene, and cis-3-hexenyl acetate were found to dominate the volatile blend (46%) in maize plants damaged by *D. maidis* females (Hill et al. 2024). The volatiles DMNT, TMTT, β-caryophyllene, β-farnesene, linalool, and methyl salicylate are associated with the profiles of infested maize plants by *D. maidis* adults (females and males) (Sanches et al. 2025). When using VOCs as a biological control strategy in maize fields, *A*. *virlai* and *P. subflava* should already be present and ready to be attracted by the VOCs. Therefore, edges and alternative habitats for egg parasitoid abundance have great importance. In addition, future field studies are needed to evaluate the efficiency of these identified VOCs in attracting *A. virlai* and *P. subflava* to *D. maidis* eggs within maize fields.

## Conclusions

The majority of *D. maidis* pest control in South America has been carried out with insecticides. However, chemical control has failed to control *D. maidis* and the corn stunt diseases transmitted by this leafhopper vector. Predators and parasitoids can be used for biological control of *D. maidis* nymphs and adults. However, limited information exists evaluating their efficiency in decreasing transmission of corn stunt disease in maize fields. A long list of native egg parasitoids has been documented in the American continents. Among these, *A. virlai* and *P. subflava* are biological control agents with especially high functional response and the ability to rapidly find *D. maidis* eggs within maize fields. They also have the highest rate of parasitism, parasitizing eggs until adult parasitoids emerge. In the present review, a methodology to control *D. maidis* populations using conservation biological control and augmentative biological control is presented. This methodology can be adapted to greenhouse or outdoor conditions. VOCs already identified may be useful to increase the efficiency of egg parasitoids under maize field conditions; the dynamics of this require investigation. Gaps in knowledge that could be investigated in future research are described in this review. Future studies are required: (i) Studies are needed to determine plant pathogen prevalence and acquisition when using predators to control *D. maidis.* In addition, dispersal of the predator community that inhabits maize edges into the maize fields surrounded by these edges could be investigated and evaluated. (ii) Adult *D. maidis* feeding behavior and dispersal when carrying plant pathogens and larval parasitoids requires investigation and characterization. (iii) Methods are needed to develop mass production of *A. virlai* and *P. subflava* using honey to increase their longevity and fecundity. (iv) The efficiency of identified VOCs in attracting *A. virlai* and *P. subflava* adults to eggs of *D. maidis* within maize fields needs to be evaluated. (v) Work on parasitoid–predator interactions would also be valuable for future studies, as predators such as spiders may also affect parasitoid populations and add complexity to parasitoid control rates.
